# Identification of Exported *Plasmodium falciparum* Proteins That Bind to the Erythrocyte Cytoskeleton

**DOI:** 10.3390/microorganisms10071438

**Published:** 2022-07-16

**Authors:** Bikash Shakya, Geoffrey Kimiti Kilili, Ling Wang, Ernesto S. Nakayasu, Douglas J. LaCount

**Affiliations:** 1 Department of Medicinal Chemistry and Molecular Pharmacology, Purdue University, West Lafayette, IN 47907, USA; bikashshaky@gmail.com (B.S.); kimjeff900@yahoo.com (G.K.K.); wang3@purdue.edu (L.W.); 2 Bindley Bioscience Center, Purdue University, West Lafayette, IN 47907, USA; ernesto.nakayasu@pnnl.gov; 3 Pacific Northwest National Laboratory, Biological Sciences Division, Richland, WA 99352, USA

**Keywords:** *Plasmodium*, exported protein, erythrocyte, cytoskeleton, red blood cell, PF3D7_1401600, ankyrin, band 4.1, host–pathogen interaction

## Abstract

*Plasmodium* proteins are exported to the erythrocyte cytoplasm to create an environment that supports parasite replication. Although hundreds of proteins are predicted to be exported through *Plasmodium* export element (PEXEL)-dependent and -independent mechanisms, the functions of exported proteins are largely uncharacterized. In this study, we used a biochemical screening approach to identify putative exported *P. falciparum* proteins that bound to inside-out vesicles prepared from erythrocytes. Out of 69 *P. falciparum* PEXEL-motif proteins tested, 18 bound to inside-out vesicles (IOVs) in two or more independent assays. Using co-affinity purifications followed by mass spectrometry, pairwise co-purification experiments, and the split-luciferase assay, we identified 31 putative protein–protein interactions between erythrocyte cytoskeletal proteins and predicted exported *P. falciparum* proteins. We further showed that PF3D7_1401600 binds to the spectrin-binding domain of erythrocyte ankyrin via its MESA erythrocyte cytoskeleton binding (MEC) motif and to the N-terminal domains of ankyrin and 4.1R through a fragment that required an intact *Plasmodium* helical interspersed sub-telomeric (PHIST) domain. Introduction of PF3D7_1401600 into erythrocyte ghosts increased retention in the microsphiltration assay, consistent with previous data that reported a reduction of rigidity in red blood cells infected with *PF3D7_1401600*-deficient parasites.

## 1. Introduction

The shape, deformability, and surface properties of host red blood cells (RBCs) are dramatically altered by infection with the malaria parasite *Plasmodium falciparum* [1,2,3]. These changes enable the parasite to prosper but contribute to pathogenesis by modifying the hemodynamic behavior of the infected RBCs (iRBCs) and altering their interactions with endothelial cells [4,5,6]. The actions of hundreds of proteins exported by the parasites to iRBCs are thought to contribute to these host cell changes. However, very little is known about the molecular interactions of these exported proteins.

More than 500 *P. falciparum* proteins are predicted to be exported [7,8,9,10]. However, the protein binding partners are known for less than a dozen [11]. Ring-infected erythrocyte surface antigen (RESA) targets spectrin [12]; knob-associated histidine rich protein (KAHRP) interacts with both spectrin and ankyrin (ANK1) [13,14,15]; mature parasite-infected erythrocyte surface antigen (MESA) has been reported to bind to 4.1R and ANK1 [16,17,18]; *P. falciparum* skeleton binding protein 1 (PfSBP1) binds to 4.1R [19] and LANCL1 [20]; *P. falciparum* erythrocyte membrane protein 1 (PfEMP1) [21] and *P. falciparum* erythrocyte membrane protein 3 (PfEMP3) [22] target actin and spectrin; lysine-rich membrane-associated PHISTb protein (LyMP/PF3D7_0532400) interacts with band 3 [23]; and *P. falciparum* protein 332 (Pf332) targets actin [24]. Similarly, we and others have demonstrated that PF3D7_0402000 binds to both 4.1R and ANK1 [25,26]. Even less is known about the functional impacts of these interactions. PfEMP1 [27] and LyMP [28] contribute to cytoadhesion of iRBCs, whereas RESA [29,30] and PfEMP3 [31] increase the rigidity and stability of the iRBC membrane, respectively. KAHRP [32] and pf332 [33,34] are involved in both membrane rigidity and adhesion of the iRBCs, which may help to provide a stable niche for the parasites to replicate inside the iRBCs in the post-capillary venules. In addition, the increased rigidity of the iRBCs also contributes to parasite sequestration and, thus, escape from destruction in the spleen [4].

A large-scale gene deletion study identified exported proteins that are likely to target the erythrocyte cytoskeleton and alter iRBC membrane function [1,2]. Since only approximately a quarter of genes encoding exported proteins were targeted for deletion in the study, it is likely that other exported proteins interact with the erythrocyte cytoskeleton and modify iRBC membrane activities. Here, we hypothesized that additional *P. falciparum* exported proteins bind to RBC cytoskeletal proteins and alter the host cell cytoskeletal membrane properties. To identify such proteins, we cloned 100 fragments from PEXEL motif-containing genes meant to mimic the processed forms exported into the host cell into a wheat germ expression vector. We successfully expressed 70 of these in small-scale in vitro translation reactions and tested their ability to bind to inside out vesicles (IOVs) prepared from uninfected RBCs. We discovered 18 exported *P. falciparum* proteins that bound to the RBC cytoskeleton in three independent assays using two distinct tags. Specific cytoskeletal binding partners were identified for 11 IOV-binding proteins using split-luciferase assays, complex purification followed by mass spectrometry, and co-purification experiments. Finally, we provided evidence that PF3D7_1401600 alters the deformability and/or stability of uninfected RBC ghosts.

## 2. Material and Methods

### 2.1. Cloning of Genes Encoding Parasite Proteins and RBC Cytoskeletal Proteins

Oligos used for cloning are listed in Supplementary Table S1. In general, genes or gene fragments encoding *P. falciparum* and human erythrocyte cytoskeleton proteins were PCR amplified using oligos with 5′ extensions homologous to sequences flanking the insertion site of the parental plasmid. Cloning was accomplished via homologous recombination in yeast. For cloning into plasmids that did not replicate in yeast, genes were amplified with oligos bearing 5′ extensions with unique restriction enzyme sites, digested with restriction enzymes, and ligated into the target plasmid with DNA ligase. One hundred and nineteen (119) genes encoding *P. falciparum* proteins containing a *Plasmodium* export element (PEXEL) or host targeting (HT) motif were amplified by RT-PCR from total RNA isolated from mixed-stage *P. falciparum* 3D7-infected RBCs using forward oligos that hybridized to sequences immediately 3′ of the predicted PEXEL/HTS site and cloned in frame with enhanced *Gaussia* luciferase (eGLuc) in plasmid p424-BYDV-eGLuc. Forty-nine (49) genes encoding parasite proteins were cloned in frame with 3XFLAG and the C-terminal fragment of firefly luciferase (C-FLuc) in plasmid p424-BYDV-CFLuc. Forty-two (42) genes or gene fragments from 22 human genes encoding RBC cytoskeletal proteins were cloned in frame with the N-terminal fragment of firefly luciferase (N-FLuc) in p424-BYDV-NFLuc. mCherry was included as a negative control and was cloned into both the N-FLuc and C-FLuc. The wheat germ expression construct pBYDV-6XHis-SBP-TEV was generated by amplifying the 6XHis and Streptavidin Binding Protein (SBP) tags from pET28-SBP-TEV (kindly provided by Dr. Alexei Savchenko, University of Calgary) and cloned into pBYDV-3XFLAG in place of 3XFLAG. Ten genes encoding parasite proteins were cloned in frame with SBP tag in pBYDV-6XHis-SBP-TEV. Four genes encoding *P. falciparum* proteins were inserted into pMAL-c4e in frame with maltose binding protein (MBP) with a hexa-histidine (6xHis) tag at the 3′ end.

### 2.2. Inside-Out Vesicle (IOV) Binding Assay

Fresh human blood was purchased from BioChemed, (Winchester, VA, USA). IOVs were prepared as described [26,35] and used for IOV-binding assays. Parasite proteins expressed in wheat germ extracts (WGE) were added to 20 μL of IOVs and the final volume was adjusted to 150 μL by adding PBS supplemented with 2% BSA and protease inhibitor (PI) cocktail. The mixture was mixed briefly and incubated on ice overnight with occasional mixing. IOVs were collected by centrifuging the mixture at 4 °C for 30 min at 3700× *g*. The resulting pellet was washed twice with cold PBS supplemented with 0.25 mM KCl, boiled in Laemmli SDS-PAGE sample loading buffer, and analyzed by Western blotting for the presence of co-purifying *P. falciparum* proteins.

### 2.3. Co-Affinity Purification of Erythrocyte Proteins That Bound to P. falciparum Proteins

Genes encoding IOV-binding proteins and eGFP were cloned in frame with the SBP tag, in vitro translated in WGE, and tested for their ability to bind with IOV, as described [26]. RBC ghosts were incubated for 2 h in 0.5 M sodium phosphate (pH 8.0), 1 M KCl plus PI cocktail on ice with occasional mixing to extract erythrocyte cytoskeletal proteins. The extract was then centrifuged at 4 °C for 30 min at 210,000× *g* to remove insoluble material and dialyzed with PBS overnight. Solubilization of erythrocyte cytoskeletal proteins was verified by SDS-PAGE followed by staining with Coommassie Blue [26].

Streptavidin-coated beads (Thermo Fisher Scientific, Waltham, MA USA, 30 μL) equilibrated by washing three times with ten volumes of SBP binding buffer (1% Triton X-100, 100 μM PMSF in PBS) were added to 15 μL SBP-tagged parasite protein, 450 μL RBC cytoskeletal extract, and 105 μL of SBP binding buffer plus PI cocktail and 100 μM PMSF. The mixture was incubated at 4 °C with constant rotation. The streptavidin beads were then collected by centrifugation, washed 3 times with 1200 μL of PBS, 0.25 M KCl, 1% Triton X-100, 100 μM PMSF, and PI cocktail with 10 min rotation per step. Proteins were eluted twice from the SBP beads by incubation with 150 μL of PBS supplemented with 2 mM biotin (Sigma-Aldrich, St. Louis, MO, USA), 1% Triton X-100, 100 μM PMSF, and PI cocktail [26,36].

### 2.4. Sample Preparation and Liquid Chromatography–Tandem Mass Spectrometry (LC–MS/MS) Analysis

Proteins from co-purification experiments and samples of soluble erythrocyte cytoskeletal protein extract were precipitated with cold acetone, digested with trypsin, and desalted using C18 microspin columns (NEST Group) as previously described [26]. Peptides were then dried by vacuum centrifugation, resuspended in 20 µL 0.1% formic acid and kept at −20 °C until LC–MS/MS analysis on an Eksigent Ekspert nanoLC 400 system connected to an AB SCIEX TripleTOF 5600 mass spectrometer. Peptides were loaded into a C18 trap column (200 µm × 0.5 mm, ChromXP C18-CL, 3 µm, 120 Å, Eksigent, Redwood City, CA, USA) and separated on a capillary C18 column (75 µm × 15 cm, ChromXP C18-CL, 3 µm, 120 Å) for 1 min in 5% solvent B (Solvent A: 0.1% FA and solvent B: 80% ACN/0.1% FA), 60 min in 5–35% solvent B, 1 min in 35–80% solvent B, 6 min in 80% solvent B, 1 min in 80–5% solvent B, and then held in 5% solvent B for 11 min at a flow rate of 200 nL/min. Eluted peptides were analyzed directly by electrospray ionization. MS scans were collected in the range of 400 to 2000 m/z. The top 50 most intense parent ions were fragmented for 50 milliseconds using rolling collision energy.

Human and *Plasmodium falciparum* 3D7 protein sequences were downloaded from UniProt Knowledge Base on 11 November 2014 in FASTA format and queried with the LC–MS/MS data using MaxQuant version 1.5.0.0 [37]. Variable modifications to the search parameters included protein N-terminus acetylation, two missed cleavages allowed, and methionine oxidation; cysteine carbamidomethylation was included as a fixed modification. The first and second search rounds used parent mass tolerances of 0.07 and 0.02, respectively. A 1% false-discovery rate was used to filter protein and peptide-spectrum matches. The significance analysis of the INTeractome (SAINT) algorithm was employed to identify proteins significantly enriched in co-purification experiments based on spectral counts [38,39].

### 2.5. Split-Luciferase Assays

RBC cytoskeletal genes cloned in frame with N-FLuc, *P. falciparum* genes encoding parasite exported proteins cloned in frame with 3X-FLAG and C-FLuc, and mCherry negative controls were in vitro translated in WGE. The levels of N-FLuc and C-FLuc proteins were normalized based on band intensities on Western blots. For binding assays, equivalent amounts of N-FLuc and C-FLuc fusion proteins were mixed in PBS containing 1% BSA plus PI cocktail, incubated for 16 h at 4 °C and assayed for luciferase activity [40]. Statistical significance was assessed using one-way ANOVA with Dunnett’s multiple comparison correction implemented in GraphPad Prism software (version 7.0d).

### 2.6. Co-Purification Assays

*P. falciparum* exported proteins were overexpressed in Novagen Rosetta (DE3) pLysS *E. coli* (Sigma-Aldrich, St. Louis, MA, USA) as fusions to MBP and His6 (MBP-His6) and affinity purified with TALON^R^ metal affinity resin (Takara Bio Clontech, San Jose, CA, USA) as described previously [35]. Bound proteins were eluted with 300 mM imidazole in PBS containing 100 μM PMSF. Co-purification experiments were performed by first binding MBP-His and MBP-His6-tagged parasite proteins to amylose beads (New England BioLabs, Ipswich, MA, USA). Samples were incubated overnight at 4 °C with rotation and washed with PBS containing 100 μM PMSF and 0.5% Triton X-100 (Haverhill, Alfa Aesar, MA, USA). Control and erythrocyte cytoskeletal proteins were in vitro translated in WGE (Promega, Madison, WI, USA) as fusions to 3X-FLAG and C-FLuc, added to the MBP-His6 proteins immobilized on amylose beads, incubated at 4 °C with rotation for 4 h, and washed three times. Samples were boiled in Laemmli SDS-PAGE sample loading buffer and subjected to Western blotting [26].

### 2.7. SDS PAGE and Western Blots

Laemmli SDS-PAGE sample buffer containing 5% β-mercaptoethanol was added to each sample. After boiling for 5 min, proteins were separated on 10% polyacrylamide gels by SDS-PAGE and transferred to nitrocellulose membranes. Membranes were probed with primary antibodies, washed, probed with HRP-conjugated secondary antibodies, and washed again. Pierce enhanced chemiluminescence (ECL) Western blotting reagent (Thermo Fisher Scientific, Waltham, MA, USA) was used to visualize antibody-labelled proteins.

### 2.8. Microsphere Filtration of MBP-His Fusion Proteins-Loaded Erythrocyte Ghosts

Erythrocyte ghosts were prepared, loaded with 30 μM MBP-His6 fusion proteins, and resealed according to previous protocols [26,41]. Briefly, washed human blood (BioChemed, Winchester, VA, USA) resuspended at 50% hematocrit in 1 mM ATP-supplemented PBS-glucose was mixed with MBP-His6-tagged parasite protein, MBP-His6, or mock-treated. The erythrocyte–protein suspension was dialyzed against hypotonic potassium buffer containing 1 mM ATP for 1 h at 4 °C in Slide-a-lyzer dialysis casettes (Thermo Fisher Scientific, Waltham, MA, USA) (3.5-kDa cut-off). Following dialysis, cells were incubated in resealing buffer for 1 h at 37 °C, washed three times in RPMI medium, and resuspended in complete GibcoRPMI medium supplemented with 4% human serum and 0.125% albumax (Thermo Fisher Scientific, Waltham, MA, USA) at 2% hematocrit. Proteinase K treatment was performed to assess loading and resealing of the RBC ghosts. Resealed cells (2.5 × 10^7^ cells) were treated with 0.5% Triton X-100, 100 μg of Proteinase K (New England BioLabs, Ipswich, MA, USA) or both and incubated for 30 min at 37 °C. PMSF was added to a final concentration of 1 mM to halt protease activity. The samples were then boiled in Laemmli SDS-PAGE sample loading buffer, separated by SDS-PAGE, and analyzed by Western blotting with anti-MBP antibody (Santa Cruz Biotechnology, Dallas, TX, USA) [26,41].

A mixture of calibrated microspheres with diameters of 5 to 15 μm and 15 to 25 μm (Type 5 and Type 6 solder powder composed of 96.50% tin, 3.00% silver, and 0.50% copper, Gesick MPM) in complete RPMI medium was loaded onto the top of a 1 mL aerosol barrier filter pipet tip (Dot Scientific, Burton, MI, USA) to create a matrix layer 4–5 mm thick above the filter [26,42]. Protein-loaded erythrocyte ghosts were passed through the microsphere matrix at a flow rate of 60 mL/h using a NE-1000electric pump (New Era Pump Systems, Farmingdale, NY, USA). The matrix was washed with 6 mL of complete RPMI medium and intact cells in the flow through were counted manually with a hemocytometer. Significance was assessed with the Mann–Whitney U test (GraphPad Prism software, version 7.0d).

## 3. Results

### 3.1. A Screen for P. falciparum-Exported Proteins That Bind to Erythrocyte IOVs

**Figure 1:**
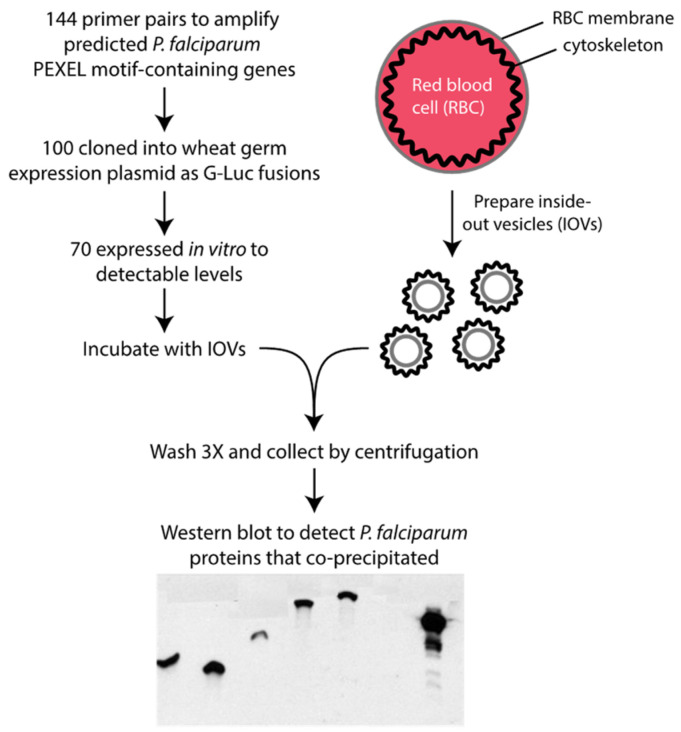
Overview of screen to identify exported *P. falciparum* proteins that bind to erythrocyte inside-out vesicles (IOVs). G-Luc, *Gaussia* luciferase.

**Figure 2:**
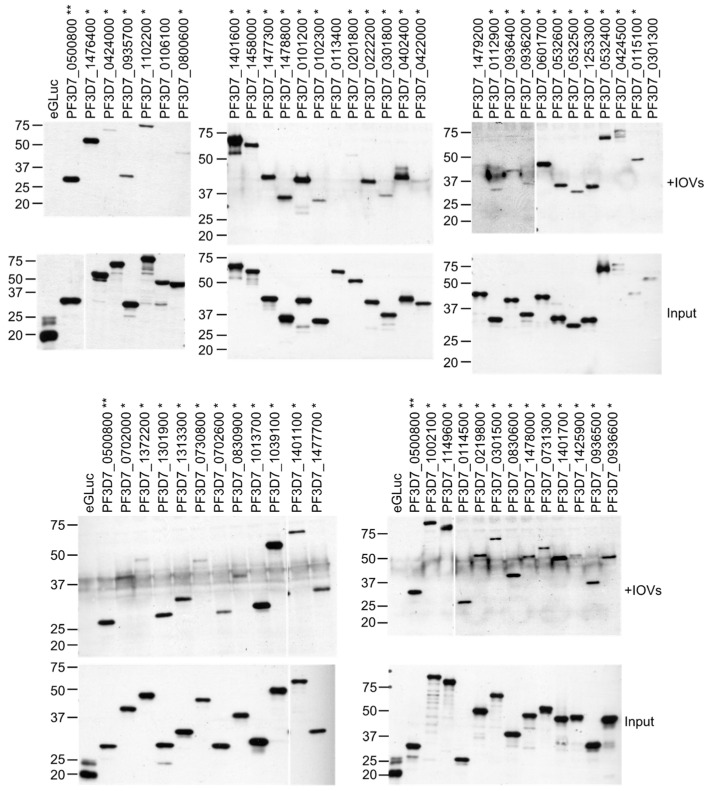
Exported *Plasmodium falciparum* proteins bound to IOVs. *P. falciparum* proteins predicted to be exported to the host cell cytoplasm were expressed in wheat germ extracts as fusions to enhanced *Gaussia* luciferase (GLuc) and incubated overnight with erythrocyte IOVs. IOVs were collected by centrifugation, washed, and subjected to SDS-PAGE and Western blotting using anti-eGLuc antibody. PF3D7_0500800 (MESA) and eGLuc were included as positive and negative controls, respectively. Molecular weight markers (sizes in kDa) are indicated at left. Blots show data from the 53 proteins that bound to IOVs in the first IOV-binding experiment. Binding was defined as the presence of a detectable band on the Western blots. * *P. falciparum* proteins that bound to IOVs ** Positive control protein PF3D7_0500800 (MESA).

To identify exported *P. falciparum* proteins that interact with components of the erythrocyte cytoskeleton or cytoplasmic domains of cell membrane proteins, we performed a large-scale biochemical screen for proteins that bound to RBC inside-out vesicles (IOVs) (Figure 1). We designed 144 pairs of primers to amplify fragments from 138 *P. falciparum* proteins predicted to be exported to the host cell via the PEXEL-dependent pathway [9,10]. The regions 3′ to the PEXEL motif sequence, which were expected to mimic the mature polypeptide exported to the host cell after processing, were targeted. Predicted exported proteins with multiple transmembrane domains were avoided due to expected problems with expression. Very large genes were divided into two or three fragments. From 144 RT-PCR reactions, 119 *P. falciparum* gene fragments were successfully amplified and 100 were cloned into p424-BYDV-eGLuc by in vivo homologous recombination in yeast [43]. Seventy (70) gene fragments from 69 genes were in vitro translated at detectable levels using wheat germ extracts (WGE) and tested in the IOV-binding assay. A fragment of PF3D7_0500800 (MESA), known to bind to the erythrocyte cytoskeleton, was included as a positive control [16]; the parental eGLuc expression plasmid with no *Plasmodium* gene insert was used as a negative control. Forty-eight protein fragments derived from 47 proteins bound to the IOVs in two independent replicates (Figure 2, Supplementary Table S2). Using data from a published *P. falciparum* gene expression study [44], we analyzed the expression of the genes encoding these IOV-binding proteins. Approximately half of the genes were maximally expressed during the first 8 h of intraerythrocytic development and 75% were expressed by 24 hpi (Supplementary Figure S1).

### 3.2. Identification of Erythrocyte Targets of IOV-Binding P. falciparum Proteins

**Figure 3:**
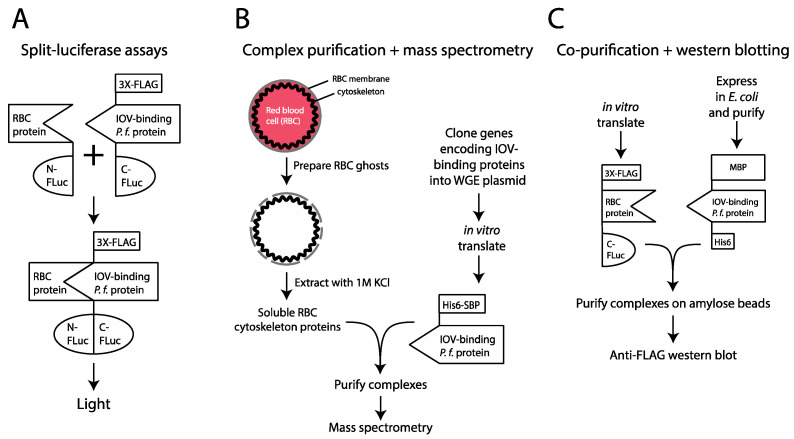
Overview of approaches to identify erythrocyte proteins targeted by IOV-binding *P. falciparum* proteins. (**A**) Split-luciferase assay. This assay is based on a pair of proteins fused to the N- and C-terminal fragments of firefly luciferase protein (N- and C-FLuc, respectively). The N- and C-FLuc fragments lack enzymatic activity and do not self-assemble into a functional enzyme. However, if the fusion partners interact, the luciferase fragments associate to form an active enzyme complex [45]. (**B**) Complex purification plus mass spectrometry. A soluble extract of erythrocyte cytoskeletal proteins was prepared by extracting RBC ghost cells with 1 M KCl and mixed with IOV-binding proteins that were in vitro translated in WGE as fusions to 6X-His and SBP tags. Complexes were purified using streptavidin beads and submitted to mass spectrometry to identify co-purifying proteins. (**C**) Co-purification was followed by Western blotting. Erythrocyte and *P. falciparum* proteins were tagged with 3X-FLAG + C-FLuc and MBP + 6X-His, respectively. *P. falciparum* proteins were expressed in *E. coli*, purified, and mixed with WGE extracts that expressed RBC cytoskeletal proteins. Complexes were purified on amylose beads and analyzed by Western blotting.

To identify the erythrocyte partners of the IOV-binding parasite proteins, we employed three complementary approaches (Figure 3). First, we tested the IOV-binding proteins for interactions with known RBC cytoskeleton and cell membrane proteins using the split-luciferase assay (Figure 3A). Genes encoding the IOV-binding proteins identified in Figure 2 were cloned into a C-FLuc expression plasmid in frame with an N-terminal 3X-FLAG epitope tag. To ensure these constructs retained their ability to interact with erythrocyte proteins, we in vitro translated the C-FLuc fusion proteins in WGE and assessed their binding to IOVs (Supplementary Figure S2). As above, the IOV-binding fragment of MESA and the C-FLuc plasmid with no *P. falciparum* insert were included as positive and negative controls, respectively. Forty-two IOV-binding proteins fused to 3X-FLAG and C-FLuc were expressed at detectable levels, of which 18 bound to IOVs (Supplementary Figure S2, Supplementary Table S2).

**Figure 4:**
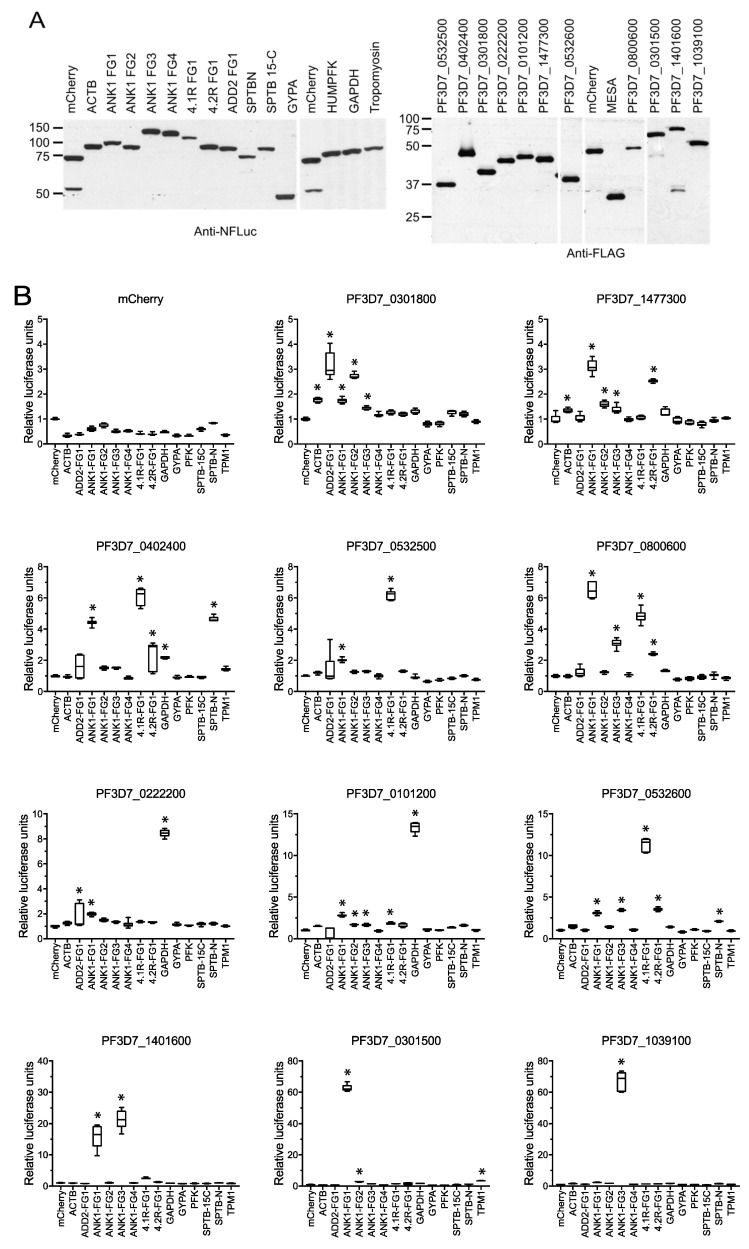
Identification of erythrocyte cytoskeletal binding partners of IOV-binding *P. falciparum* proteins. Full length human erythrocyte cytoskeleton proteins and protein fragments were expressed in wheat germs extracts as fusions to the N-terminus of firefly luciferase (N-FLuc). Similarly, IOV-binding *P. falciparum* proteins were expressed as fusions to the C-terminal domain of firefly luciferase (C-Fluc). (**A**) Equivalent levels of N-FLuc (**left**) and C-FLuc (**right**) fusion proteins were verified by Western blotting using anti-N-FLuc and anti-FLAG tag antibodies. mCherry was expressed as fusions to N-FLuc and C-FLuc and was included as a negative control. Molecular weight markers (sizes in kD) are indicated at the left of each blot. (**B**) Relative luciferase activity (RLA) from pairs of erythrocyte cytoskeleton and *P. falciparum* proteins. Box and whisker plots show the average of the six luciferase readings normalized to the luciferase activity of each *P. falciparum* protein with N-FLuc-mCherry, which was set to 1. Whiskers indicate the largest and smallest values. Asterisks indicate interactions that produced significantly greater RLA than the N-FLuc-mCherry negative control. * *p* ≤ 0.001; ACTB, β-actin; ANK1, Ankyrin 1; 4.1R, Band 4.1; 4.2R, Band 4.2; GYPA, glycophorin A; GAPDH, glyceraldehyde phosphate dehydrogenase; SPTB-N, N-terminal fragment of β-spectrin; SPTB 15-C, β-spectrin fragment spanning spectrin repeat 15th to the C-terminus; ADD2, β-adducin; PFK, phosphofructokinase; TPM1, Tropomyosin.

A panel of RBC cytoskeletal proteins was generated to test for interactions with the IOV-binding parasite proteins. Forty-two (42) gene fragments from 22 RBC cytoskeletal proteins or cytoplasmic domains of cell membrane proteins were cloned into the N-FLuc expression plasmid (Supplementary Figures S3 and S4). N- and C-FLuc-tagged proteins were in vitro translated in WGE and their concentrations adjusted so that the test proteins were present in equal or slightly lower amounts relative to the negative controls. All combinations of N- and C-FLuc-tagged proteins were tested for luciferase activity [40]. mCherry cloned into the N-and C-FLuc expression plasmids was included as a negative control. In our initial screen, 12 parasite proteins and 14 erythrocyte protein fragments produced ≥5-fold increase in relative luciferase activity (RLA) with at least one partner, indicating they were functional in the split-luciferase assay. All pairwise combinations of these constructs were then re-tested in triplicate (Figure 4B). A total of 40 interactions involving 11 parasite proteins and 10 erythrocyte protein fragments yielded significantly higher RLA compared to negative controls (*p* < 0.001) (Figure 4B, Table 1).

### 3.3. Co-Precipitation of Cytoskeletal Proteins from Erythrocyte Lysates with IOV-Binding P. falciparum Proteins

To provide an independent assessment of the RBC cytoskeletal proteins targeted by the IOV-binding proteins, we used mass spectrometry to characterize complexes formed with exported *P. falciparum* proteins (Figure 3B). Nine IOV-binding parasite proteins were in vitro translated as fusions to SBP and incubated with soluble cytoskeletal proteins extracted from erythrocyte ghosts by incubation with 1 M KCl. As negative controls, we included SBP-tagged GFP and SBP-PF3D7_0936400, a predicted exported protein that did not bind to IOVs. Complexes were purified on streptavidin beads, washed, eluted with biotin and subjected to LC–MS/MS. SBP-GFP, SBP-PF3D7_0936400, the positive control MESA (PF3D7_0500800), and eight IOV-binding parasite proteins identified in this study (PF3D7_1401600, PF3D7_0402400, PF3D7_0936400, PF3D7_1039100, PF3D7_0532600, PF3D7_0800600, PF3D7_0101200, and PF3D7_0301500) were detected in their respective pull-down experiments, confirming the bait protein was successfully purified (Table 2). Proteomic analysis of the KCl extract erythrocyte ghosts identified 270 proteins, with the most abundant being erythrocyte cytoskeleton proteins (Supplementary Table S3).

LC–MS/MS data was analyzed using the SAINT program to distinguish true interactions from the non-specific background [38]. In total, 59 erythrocyte proteins were detected at significantly higher levels in co-purification experiments with IOV-binding proteins compared to SBP-GFP negative control samples (SAINT score > 0.9, Table 2 and Supplementary Table S4). However, 33 of the 59 proteins were common contaminants in the CRAPome database (version 1.1) and are unlikely to represent true binding partners (Supplementary Table S4) [46]. ANK1 was the most frequently detected cytoskeletal protein, co-purifying with PF3D7_1401600, PF3D7_0500800, and PF3D7_1039100. Dematin, spectrin-α, and spectrin-β also co-purified with at least one *P. falciparum* protein (Supplementary Table S4).

### 3.4. Confirmation of Protein–Protein Interaction in Co-Precipitation Assays

**Figure 5:**
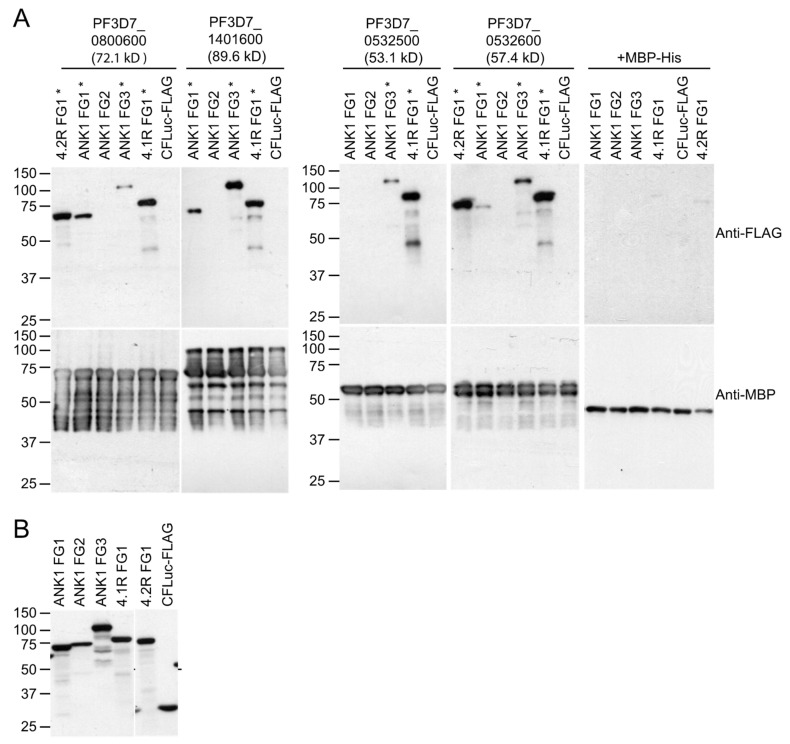
Co-precipitation of erythrocyte cytoskeletal proteins with *P. falciparum*-exported proteins. (**A**) MBP-His-tagged *P. falciparum* proteins were incubated with human RBC cytoskeleton proteins tagged with 3X-FLAG and C-FLuc, purified on amylose beads, and analyzed by SDS-PAGE and Western blotting with anti-FLAG (top panel) and anti-MBP (bottom panel) antibodies. The 3XFLAG + C-FLuc and MBP-His construct without human or *P. falciparum* inserts, respectively, were included as negative controls. Molecular weight markers (sizes in kDa) are indicated at left. The expected sizes of the parasite proteins are shown in parentheses. * indicates erythrocyte proteins that co-purified with the specified bait protein. (**B**) Western blot showing input amounts of human RBC proteins. Molecular weight markers (sizes in kDa) are indicated at left.

**Figure 6:**
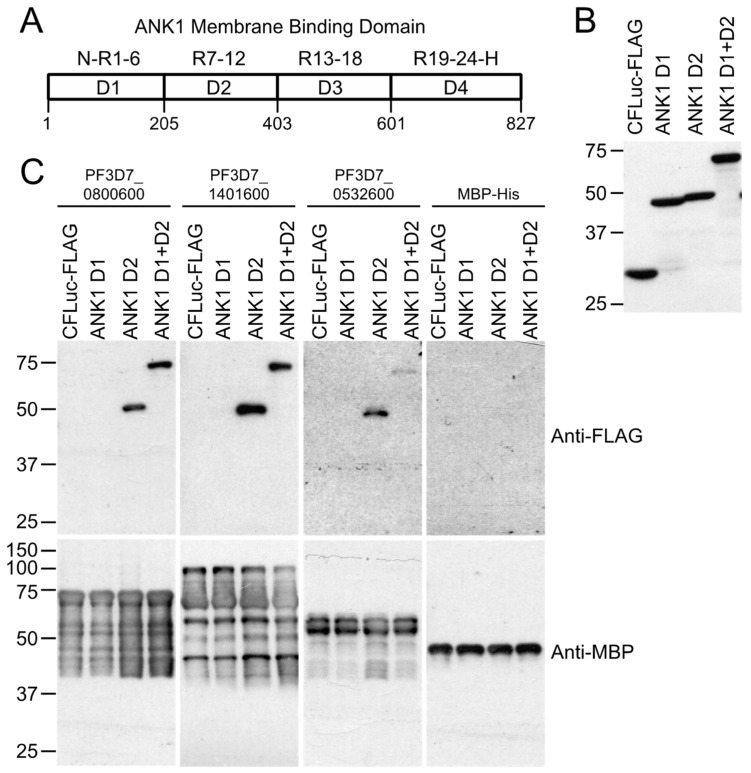
Exported *P. falciparum* proteins bind to the second folded domain of the membrane binding domain (MBD). (**A**) The ANK1 MBD consists of four folded domains: D1, N- terminus through ankyrin repeat 6 (N-R1-R6); D2, ankyrin repeats 7–12 (R7–R12); D3, ankyrin repeats 13–18 (R13–R18); D4, ankyrin repeats 19–24 plus the hinge region (R9–R24-H). Numbers indicate amino acid positions. (**B**) Expression of C-FLuc + 3XFLAG tagged ANK1 domains. D1, D2, and D1 + D2 domains were in vitro translated and subjected to Western blotting with anti-FLAG tag antibody. Molecular weight markers (sizes in kDa) are indicated at left. (**C**) Co-affinity purification of D1 and D2 domains of ANK1 MBD with exported *P. falciparum* proteins that bound to ANK1 fragment 1. C-FLuc-3XFLAG-tagged ANK domains were incubated with MBP-HIS-tagged parasite proteins, purified with amylose resin, and subjected to SDS-PAGE analysis and Western blotting using anti-FLAG (**top**) and anti-MBP antibodies (**bottom**). Molecular weight markers (sizes in kD) are indicated at left.

To confirm the protein–protein interactions observed in the split-luciferase and co-affinity/mass spectrometry experiments, we performed co-precipitation assays with proteins synthesized in WGE or *E. coli* (Figure 3C). PF3D7_0800600, PF3D7_1401600, PF3D7_0532600, and PF3D7_0532500 were successfully expressed in *E. coli* as fusions to the MBP and 6His tags (MBP-His6). These proteins were purified and incubated with in vitro translated FLAG and C-FLuc-tagged erythrocyte cytoskeletal protein fragments. Of 12 interactions identified for these four *P. falciparum* proteins in the assays described above, 11 were confirmed by the co-precipitation assays (Figure 5, lanes marked by asterisks). MBP-His6 did not co-precipitate any erythrocyte protein and the negative control did not co-purify with any MBP-His-tagged proteins. Interactions between PF3D7_1401600 and 4.1R and between PF3D7_0532500 and ANK1 FG3 were identified in the co-precipitation experiments but not in the split-luciferase assay (Figure 5 and Table 1). ANK1 was the most common partner for exported proteins tested in this study, interacting with 11 proteins in the split-luciferase assay and all 4 proteins tested in co-purification assays. In particular, the N-terminal half of the ANK1 membrane binding domain (MBD) appears to be a major target. The MBD consists of 24 ankyrin repeats arranged in four folded domains (D1–4) [47]. To further define the region in the ANK1 targeted by the *P. falciparum*-exported proteins, additional co-precipitation assays using the D1 and D2 domains were performed. PF3D7_0800600, PF3D7_1401600, and PF3D7_0532600 bound to the D2 domain, indicating the ANK1 binding sites are within ankyrin repeats 7 through 12 (Figure 6).

### 3.5. Mapping the Minimum Binding Domain of PF3D7_1401600 for ANK1 and 4.1R

**Figure 7:**
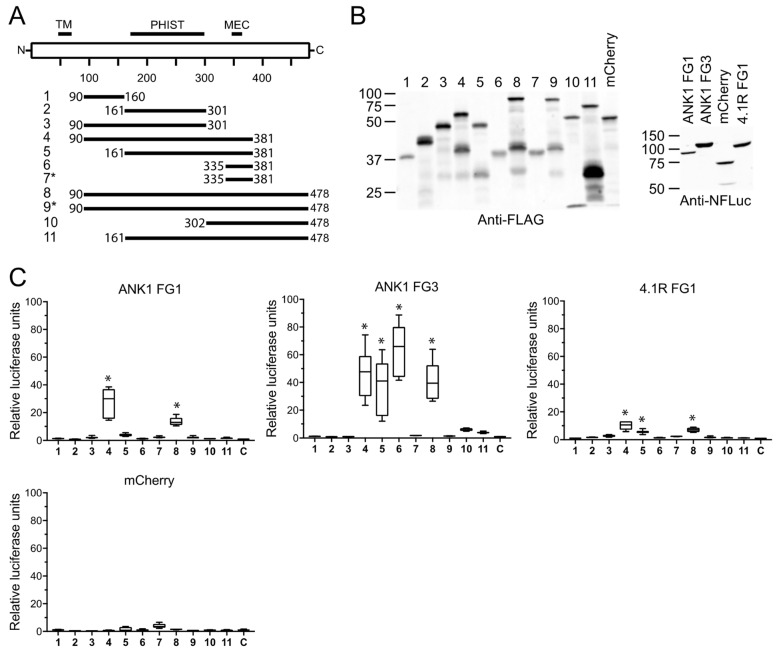
Mapping the protein interaction domains of PF3D7_1401600. (**A**) PF3D7_1401600 deletion constructs (solid bars) were used to define the regions that interacted with ANK1 FG1, ANK1 FG3, and 4.1R FG1. TM indicates predicted transmembrane domain; MEC, MESA erythrocyte cytoskeleton binding domain; PHIST, poly-helical interspersed sub-telomeric domain. Numbers indicate amino acid positions. Asterisks indicate fragments in which amino acids YID in the MEC motif were changed to AAA. (**B**) Expression of N-FLuc and C-FLuc fusion proteins. Eleven fragments of PF3D7_1401600 and mCherry-C-FLuc were expressed as fusion to 3X-FLAG and C-FLuc and subjected to Western blotting with anti-FLAG antibody (**left** panel). Similarly, ANK1 FG1, ANK1 FG3, 4.1R FG1, and mCherry were expressed in WGE as fusion to N-FLuc and subjected to Western blotting with anti-N-Fluc antibody (**right** panel). Molecular weight markers (sizes in kD) are shown to the left of each blot. (**C**) Relative luciferase activity of N-Fluc-ANK1 FG1, -ANK1 FG3, and -4.1R FG1 following incubation with C-FLuc-PF3D7_1401600 fragments. Luciferase values were normalized to the activity of the RBC protein with C-FLuc-mCherry, which was set at 1. Whiskers indicate the largest and smallest values. Asterisks indicate combinations that yielded luciferase activity significantly greater than both negative controls (* *p* < 0.05).

PF3D7_1401600 contains an MEC motif and a PHIST domain [2,9,35]. Since PF3D7_1401600 interacted with two fragments of ANK1 (FG1 and FG3) and an N-terminal fragment of 4.1R in split-luciferase and co-precipitation assays (Figure 4 and Figure 5), we sought to determine if the interactions were mediated by different regions of PF3D7_1401600. To identify the minimal binding regions, eleven deletion constructs of PF3D7_1401600 were tested in the split-luciferase assay (Figure 7).

Constructs 4, 5, 6, and 8 displayed a significantly increased luciferase activity when paired with ANK1 FG3. The smallest PF3D7_1401600 construct (fragment 6) that interacted with ANK1 FG3 consisted of amino acids 335 to 381, which includes the MEC motif (Figure 7C, middle panel). Constructs that lacked the MEC motif (1, 2, and 3) or that contained a three-residue mutation that disrupted the binding to ankyrin (YID to AAA, constructs 7 and 9, [18]) did not interact with ANK1 FG3 (Figure 7C). Constructs 10 and 11, which included the MEC motif, had higher relative luciferase values than fragments lacking or bearing a mutated MEC motif (1, 2, 3, 7, and 9; 4- and 6-fold relative to the negative control versus 1.8-fold or less), but did not reach statistical significance. Together, these data suggest the MEC motif is sufficient to mediate binding to ANK1 FG3.

In contrast to ANK1 FG3, the PF3D7_1401600 fragments that interacted with ANK1 FG1 and 4.1R FG1 did not reveal well-defined binding regions. ANK1 FG1 had a significantly elevated luciferase activity with fragments 4 and 8, whereas 4.1R FG1 showed significant increases with fragments 4, 5, and 8. All three fragments contain the PHIST domain and the MEC motif, but constructs consisting solely of the PHIST domain or MEC motif (fragments 2 and 6, respectively) did not yield increased luciferase activity. Comparing fragments 4 and 5 suggested that ANK1 FG1 required amino acids 90–160 for binding. However, ANK1 FG1 did not interact with fragment 1, which corresponds to this region of PF3D7_1401600. It is not clear from these data if the minimal binding domains overlap with the junctions between fragments or if the smaller fragments are non-functional in the split-luciferase assay.

### 3.6. Effect of Binding to the RBC Cytoskeleton on the Mechanical Properties of Erythrocyte Ghosts

Binding of *P. falciparum*-exported proteins to RBC cytoskeletal proteins can affect erythrocyte membrane rigidity and stability [2,31,48,49]. To investigate the effect of PF3D7_1401600, PF3D7_0532600 and PF3D7_0532500 on the RBC cytoskeleton, full length proteins (minus the region N-terminal to the PEXEL motif) were expressed in *E. coli*, purified, and loaded into erythrocyte ghosts, which were then analyzed in the microsphiltration assay. This approach uses metal microspheres to simulate inter-endothelial splenic slits and the mechanical challenges encountered by infected RBCs as they pass through the spleen [26,29,42]. Changes in erythrocyte deformability are detected by differences in the efficiency of passage through the matrix.

**Figure 8:**
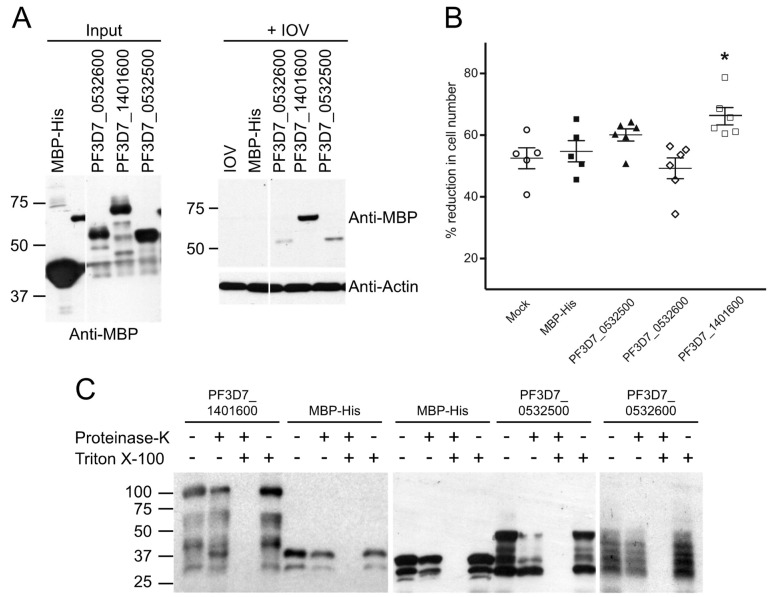
PF3D7_1401600 reduced the deformability of erythrocyte ghosts. (**A**) IOV binding assay. Purified MBP-His and MBP-His-*P. falciparum*-exported proteins were incubated with erythrocyte IOVs, washed with binding buffer, and subjected to SDS-PAGE analysis followed by Western blotting with anti-MBP antibody. Molecular weight markers (sizes in kDa) are indicated at left. (**B**) Microsphiltration of ghost erythrocytes loaded with *P. falciparum* proteins. Resealed ghost cells were passed through a microsphere matrix to assess the effect of parasite proteins on erythrocyte deformability. The number of intact cells in starting and flow-through samples were counted on a hemocytometer and used to calculate the percent reduction in post-column sample. Statistical significance was assessed using the Mann–Whitney U test (* *p* < 0.05). (**C**) Proteinase protection assay. Erythrocyte ghosts were loaded with mock or MBP-His or MBP-His-tagged parasite proteins (30 μM each) and mock treated (indicated by -) or treated with 100 μg/mL of Proteinase K, 0.5% Triton X-100, or both (indicated by +). The treated cells were subjected to Western blotting using anti-MBP antibody.

Purified and dialyzed MBP-His-tagged parasite proteins or MBP-His were loaded into erythrocytes at a concentration of 30 μM and resealed [12,26,31,41]. At this concentration, the amount of protein loaded into the resealed ghosts was predicted to be in excess of 4.1R and ANK1 [26]. IOV-binding of the MBP-His-tagged parasite proteins was verified prior to loading (Figure 8A). Successful loading of the proteins into the ghosts was confirmed by protection of the loaded proteins from degradation by proteinase K (Figure 8C) [26,41]. After loading, the resealed ghosts were passed through the microsphere matrix and the amount of intact red blood cells in the flow-through medium was compared to the pre-column sample. Ghost cells loaded with PF3D7_1401600 were recovered at significantly lower levels compared to MBP-His- or mock-loaded ghosts (Figure 8B), suggesting that PF3D7_1401600 affected erythrocyte cytoskeletal function, leading to reduced deformability or increased cell lysis. No effect was observed for the negative control, MBP-His, or the exported proteins PF3D7_0532600 and PF3D7_0532500, possibly due to the lower levels of binding to IOVs relative to PF3D7_1401600 (Figure 8A).

## 4. Discussion

**Figure 9:**
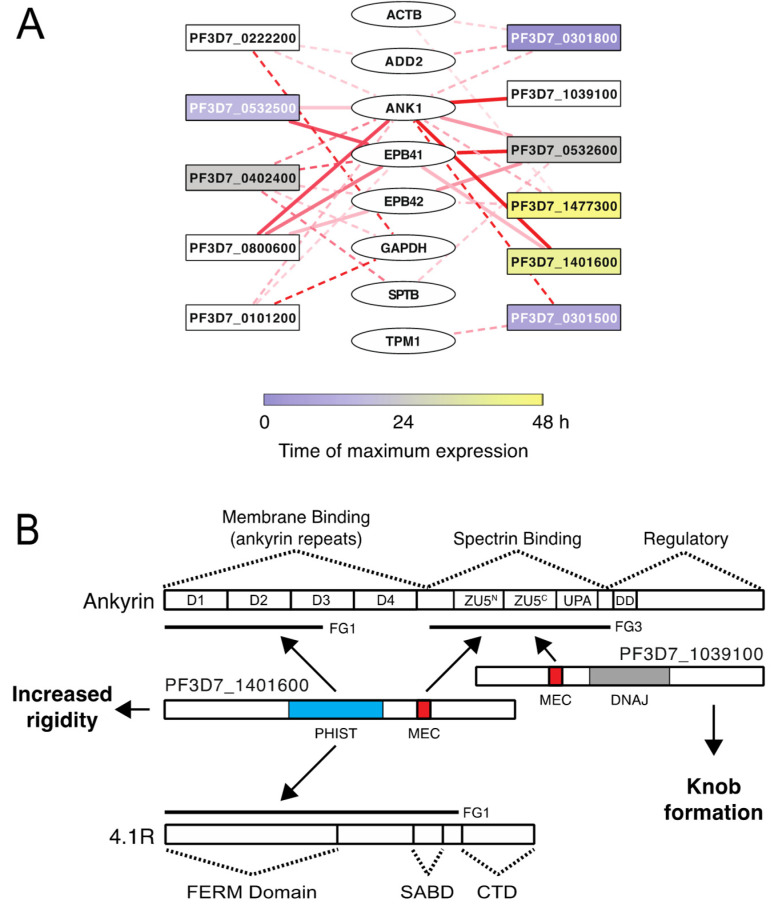
(**A**) Summary of interactions between exported *P. falciparum* proteins (rectangles) and erythrocyte cytoskeleton proteins (ovals). Lines represent protein–protein interactions. Dashed lines indicate interactions identified only by the split-luciferase assay. Solid lines indicate interactions found in the split luciferase assay and in co-affinity purification experiments. The intensity of the red color corresponds to the strength of the luciferase signal with dark red indicating the strongest signal. Shading of the rectangles indicates the time of maximum RNA expression during the erythrocytic life cycle. (**B**) Model for the interaction of MEC motif proteins PF3D7_1401600 and PF3D7_1039100 with erythrocyte ankyrin and 4.1R. PF3D7_1401600 and PF3D7_1039100 bind to the spectrin binding domain of ANK1 via the MEC motif. PF3D7_1401600 makes additional contacts with ANK1 and 4.1R through the PHIST domain, which may account for its effect on membrane rigidity. PF3D7_1039100 does not bind to other regions of ANK1 or 4.1R and does not affect rigidity. In contrast, PF3D7_1039100 is required for knob formation, possibly through interactions with other exported *P. falciparum* proteins. D1–D4, independently folded ankyrin repeat subdomains; ZU5, domain present in ZO-1 and Unc5-like netrin receptors, N and C refer to N- and C-terminal copies of ZU5 domain; UPA, domain conserved in UNC5, PIDD, and Ankyrins; DD, death domain; FERM, Four-point-one, Ezrin, Radixin, Moesin domain; FA, FERM adjacent domain; SAB, Spectrin-Actin Binding domain; CTD, C-terminal domain; DNAJ, DnaJ molecular chaperone homology domain.

Hundreds of *P. falciparum* proteins are predicted to be exported to the erythrocyte during infection, representing a large investment by the parasite to manipulate the host cell. Although the process of protein export is essential for parasite replication [50], the functions of most *P. falciparum*-exported proteins have not been characterized, apart from a few proteins such as RESA, PfEMP3, KAHRP, and MESA [12,13,16,17,22,31,51,52,53]. Here, we report the binding of 47 predicted exported *P. falciparum* proteins to the erythrocyte cytoskeleton (Figure 2 and Supplementary Table S2). Of the 70 fragments from 69 proteins that were successfully expressed in WGE, 48 bound to inside out vesicles in at least one assay, and 18 bound in two or more independent assays. Using a combination of co-affinity purification from erythrocyte lysates followed by mass spectrometry, co-purifications with purified proteins and pairwise split-luciferase assay, we identified 31 putative direct interactions between exported *P. falciparum* proteins and erythrocyte cytoskeletal proteins (Table 1 and Figure 9A). Genes encoding 7 out of 18 proteins from the highest confidence group (i.e., those that bound to IOVs as fusions to two different protein tags) were refractory to transposon insertion [54,55], suggesting they are essential for parasite growth (Supplementary Table S2). The non-essential genes may encode proteins that are required for growth in vivo or may have redundant functions with other exported proteins.

ANK1, and to a lesser extent 4.1R, were the major targets of the exported proteins identified here. Both play prominent roles in linking the spectrin cytoskeleton to the cell membrane. Among the proteins that bound to ANK1 were PF3D7_1401600, which contributes to the increased rigidity of infected erythrocytes, and PF3D7_1039100, which is required for knob formation on the surface of infected RBCs [2]. Both PF3D7_1401600 and PF3D7_1039100 contain an MEC motif (Figure 9B) [35] and bind to ANK1-FG3, which includes the spectrin-binding domain of ANK1 (Supplementary Figure S3). In a separate study of the binding partners of the MESA MEC motif, we demonstrated that the MEC binding site in ANK1 was C-terminal to the spectrin-binding site and therefore unlikely to compete with spectrin for binding [18]. In both PF3D7_1401600 and MESA, the MEC motif was sufficient for binding to ANK1 fragment 3 (Figure 7 and [18]).

In addition to ANK1-FG3, PF3D7_1401600 also bound to ANK1-FG1 and to 4.1R-FG1 through an MEC-independent site that contains the PHIST domain (Figure 9B). Since deletion of *PF3D7_1401600* reduced erythrocyte membrane rigidity [2], we tested the effect of PF3D7_1401600 on RBC membrane properties. Erythrocyte ghosts loaded with purified PF3D7_1401600 passed through a matrix of metal microspheres less efficiently than mock-loaded erythrocytes or erythrocytes loaded with MBP-His (negative control that does not bind to the erythrocyte cytoskeleton), PF3D7_0532500 (binds to ANK1-FG1 and 41.R), or PF3D7_0532600 (binds ANK1-FG1 and -FG3, 4.1R, 4.2R, and β-spectrin). Thus, PF3D7_1401600 appears to increase membrane rigidity or reduce stability when introduced individually into erythrocytes. Together, these data strongly suggest that the function of PF3D7_1401600 is to regulate flexibility of the infected RBC membrane through its interactions with the N-terminal region of ANK1 and/or 4.1R (Figure 9B).

As with PF3D7_1401600, the PHIST domain proteins PF3D7_0800600 and PF3D7_0402000 also bound to ANK1-FG1 and to 4.1R-FG1 (Figure 4 and Figure 5, and [26]). In the case of PF3D7_0402000, loading of purified protein into erythrocyte ghosts reduced the number of intact cells that passed through a microsphiltration column, either due to increased retention or increased lysis [26]. It is not clear if this effect requires the PHIST domain-mediated interactions with ANK1, 4.1R, or both. The binding sites for PF3D7_1401600 and PF3D7_0402000 in ANK1-FG1 were localized to ankyrin repeats 6–12 (also known as domain 2 or D2), which is the same region that binds to band 3. Similarly, 4.1R-FG1 also binds to band 3. Disrupting the interactions of band 3 with 4.1R or ANK1 increased the rigidity [56] and decreased the stability [57] of the RBC membrane, respectively. Together, these observations suggest that binding of PF3D7_0402000, PF3D7_0800600, and PF3D7_0402000 to 4.1R and ANK1 may alter erythrocyte rigidity by affecting the binding properties of band 3, specifically by interfering with band 3 binding to 4.1R and/or by stabilizing band 3 binding to ANK1. However, the net impact of these interactions in *P. falciparum*-infected erythrocytes is likely to be complex due to the numerous exported proteins that have similar binding interactions with the RBC cytoskeleton.

In contrast to PF3D7_1401600, PF3D7_1039100 lacks a PHIST domain and is bound only to ANK1-FG3. This interaction is predicted to be mediated by its MEC motif, though we did not perform a deletion analysis to test this. Consistent with the lack of interactions with ANK1-FG1 and 4.1R-FG1, deleting *PF3D7_1039100* had no effect on membrane rigidity in infected cells [2]. However, parasites lacking *PF3D7_1039100* had reduced knob formation [2]. Since no other erythrocyte cytoskeleton interactions were identified for PF3D7_1039100, its effect on knob formation may be mediated through interactions with other parasite proteins.

Pf3D7_0222200 and its paralog PF3D7_0101200 belong to the PfEPF3 (Hyp4) family of exported proteins [58]. Both bound to IOVs and to GAPDH in the split-luciferase assay. GAPDH is associated with the erythrocyte cytoskeleton primarily through its interaction with the N-terminus of band 3 [59,60], but is also bound to actin, ANK1, spectrin protein 4.2, and p55 in photocross-linking experiments [61]. Biochemical fractionation and localization studies indicated that PfEPF3 family members are peripheral membrane proteins that associate with Maurer’s clefts [58].

## 5. Limitations

We acknowledge the potential for both false positives and false negatives in our approach. Although great care was taken to reduce nonspecific binding, we cannot completely eliminate this possibility. Since there is still uncertainty in the prediction of exported proteins, some of the proteins identified as IOV-binders may not actually be exported; confirmation of export in infected cells will be an important next step. Experiments to identify direct binding partners can also be difficult to interpret. For example, although α- and β-spectrin co-purified with both MESA and PF3D7_1401600 in co-precipitation assays from erythrocyte lysate (Table 2), the interactions were not recapitulated in the split-luciferase assay. These could be indirectly mediated via ANK1, which forms a protein complex with spectrin in the erythrocyte cytoskeleton [62]. On the other hand, all protein–protein interaction assays have high false-negative rates and some interactions can only be identified using particular methods [63]; it is likely that some interactions have been missed due to technical limitations in the split-luciferase and co-affinity purification assays, or because the assays were performed with single parasite proteins and IOVs or lysates from uninfected RBCs.

Our study was limited to a subset of PEXEL-motif proteins and thus did not exhaustively analyze all exported *P. falciparum* proteins. PEXEL/HT-negative exported proteins (PNEPs) [64,65] and PEXEL-containing genes that were not included in our initial list or that could not be amplified, cloned, or expressed were not evaluated. Finally, our resealed erythrocyte ghost cell assays incorporated a single *P. falciparum* protein, and therefore would miss any combinatorial effects from the hundreds of proteins predicted to be exported during infection.

## 6. Conclusions

This study revealed 47 putative IOV-binding proteins and identified 31 potential direct interactions with erythrocyte cytoskeletal proteins. These interactions help to explain the functions of exported proteins PF3D7_1401600 (increased rigidity of iRBC), PF3D7_1039100 (knob formation), and the PfEPF3 family members Pf3D7_0222200 and PF3D7_0101200 (Maurer’s clefts) and will serve as the basis for detailed follow up experiments.

## Figures and Tables

**Table 1:** Summary of interactions between erythrocyte cytoskeletal and exported *P. falciparum* proteins.

*P. falciparum* Protein	Erythrocyte Protein	Erythrocyte Protein Fragment	Split-Luciferase	Co-Purification/Western	Co-Purification/Mass Spec
PF3D7_0101200	ANK1	FG1, 2, 3	+		
	EPB41	FG1	+		
	GAPDH	-	+		
PF3D7_0222200	ADD2	FG1	+		
	ANK1	FG1	+		
	GAPDH	-	+		
PF3D7_0301500	ANK1	FG1, 2	+		
	TPM1	-	+		
PF3D7_0301800	ACTB	-	+		
	ADD2	FG1	+		
	ANK1	FG1, 2, 3	+		
PF3D7_0402400	ANK1	FG1	+		
	EPB41	FG1	+		
	EPB42	FG1	+		
	GAPDH	-	+		
	SPTB	N	+		
PF3D7_0532500	ANK1	FG1	+		
	ANK1	FG3		+	
	EPB41	FG1	+	+	
PF3D7_0532600	ANK1	FG1, 3	+	+	
	EPB41	FG1	+	+	
	EPB42	FG1	+	+	
	SPTB	N	+		
PF3D7_0800600	ANK1	FG1, 3	+	+	
	EPB41	FG1	+	+	
	EPB42	FG1	+	+	
PF3D7_1039100	ANK1	FG3	+		+
PF3D7_1401600	ANK1	FG1, 3	+	+	+
	EPB41	FG1		+	
PF3D7_1477300	ACTB	-	+		
	ANK1	FG1, 2, 3	+		
	EPB42	FG1	+		

**Table 2:** Identification of erythrocyte proteins bound to exported *P. falciparum* proteins by co-affinity purification plus mass spectrometry.

Bait Protein	Proteins Detected by Mass Spectrometry	Gene Symbol	Spectral Counts *	Control Spectral Counts *	Fold Enrichment **	Saint Score	BFDR ***
PF3D7_1401600	*Plasmodium*-exported protein (PHISTb), unknown function (PF14_0018)	PF3D7_1401600	140|91	0|0	1155	1	0
	Ankyrin 1	ANK1	117|56	0|0	865	1	0
	Spectrin alpha, erythrocytic 1	SPTA1	96|45	0|0	705	1	0
	spectrin beta, erythrocytic	SPTB	72|28	0|0	500	1	0
PF3D7_0402400	*Plasmodium*-exported protein, unknown function (PFD0115c)	PF3D7_0402400	77|41	0|0	590	1	0
	KRAS proto-oncogene, GTPase; HRas proto-oncogene, GTPase; NRAS	KRAS;HRAS;NRAS	2|3	0|0	25	1	0
PF3D7_0936400	Ring-exported protein 4 (REX4)	REX4	73|41	0|0	570	1	0
PF3D7_1039100	DNAJ protein, putative (PF10_0381)	PF3D7_1039100	78|18	0|0	480	1	0
	Ankyrin 1 (ANK1)	ANK1	18|5	0|0	115	1	0
PF3D7_0532600	*Plasmodium*-exported protein, unknown function (PFE1615c)	PF3D7_0532600	43|36	0|0	395	1	0
PF3D7_0800600	*Plasmodium*-exported protein (PHISTa), unknown function (MAL8P1.163)	PF3D7_0800600	20|68	0|0	440	1	0
PF3D7_0101200	*Plasmodium*-exported protein (hyp4), unknown function (PFA0060w)	PF3D7_0101200	24|15	0|0	195	1	0
PF3D7_0500800	*Plasmodium* mature parasite-infected erythrocyte surface antigen (MESA)	MESA	99|91	0|0	950	1	0
	Ankyrin 1	ANK1	405|525	0|0	4650	1	0
	Spectrin alpha, erythrocytic 1	SPTA1	39|24	0|0	315	1	0
	Spectrin beta, erythrocytic	SPTB	24|19	0|0	215	1	0
	Dematin actin binding protein	DMTN	3|3	0|0	30	1	0
PF3D7_0301500	*Plasmodium*-exported protein, unknown function (PFC0075c)	PF3D7_0301500	26|19	0|0	225	1	0

* Spectral counts from two replicates (Replicate1|Replicate2). ** Fold enrichment was calculated by dividing the average spectral counts from the MBP-His-tagged bait protein co-purification by that of the MBP-His co-purification. If the prey protein was not detected in the MBP-His co-purification, the average spectral counts from the bait protein co-purification was divided by 0.1. *** BFDR, Bayesian false discovery rate.

## Data Availability

Data is contained within this article and the Supplementary Tables.

## Supplementary Materials

The following supporting information can be downloaded at: https://www.mdpi.com/article/10.3390/microorganisms10071438/s1, Figure S1: Expression pattern of *P. falciparum* genes encoding IOV-binding proteins; Figure S2: Binding of *P. falciparum* exported proteins to inside out vesicles (IOVs); Figure S3: Domain organization of erythrocyte cytoskeletal protein fragments used in the split-luciferase assay; Figure S4: Domain organization of *P. falciparum* exported proteins that bound to erythrocyte cytoskeletal proteins; Table S1. List of oligos in the study; Table S2. Summary of screen for exported *P. falciparum* proteins that bind to IOVs; Table S3. Proteins identified in soluble erythrocyte cytoskeleton extract; Table S4. Comparison of mass spectrometry results to common contaminants in the CRAPome database.
