# Supplementary material for: Identification of Exported Plasmodium falciparum Proteins That Bind to the Erythrocyte Cytoskeleton
Source: Microorganisms. 2022 Jul 16;10(7):1438. doi: 10.3390/microorganisms10071438 (PMC9320996; doi:10.3390/microorganisms10071438)

Shakya et al.

Supplementary Figure Legends

Supplementary Fig. S1. Expression pattern of *P. falciparum* genes encoding IOV-binding proteins. RNA-Sequencing data from Otto et al. [46] were used to construct a heat map showing changes in transcript levels at seven time points in the parasite life cycle. RNA-Sequencing data in the form of geometric means was converted to Z-scores and a heat map was constructed in Microsoft Excel. Forty (40) genes encoding IOV-binding proteins from Fig. 1 were present in the data from Otto et al.

Supplementary Fig. S2. Binding of *P. falciparum* exported proteins to inside out vesicles (IOVs). Western blots of IOV binding assay for *P. falciparum* exported proteins. *P. falciparum* proteins were expressed as fusions to the C-FLuc and 3X-FLAG tags in wheat germ extract (WGE) and were separately incubated overnight with same amount of erythrocyte IOVs, collected by centrifugation, washed, and subjected to SDS-PAGE analysis and western blotting using anti-FLAG antibody. PF3D7_0500800 (MESA) and eGFP were included as positive and negative controls, respectively. Molecular weight markers (sizes in kDa) are indicated at left.

Supplementary Fig. S3. Domain organization of erythrocyte cytoskeletal protein fragments used in the split-luciferase assay. Human erythrocyte proteins are represented as rectangular bars whose length is proportional to the size in amino acids, as indicated by the scale bar. Black lines represent the fragments of the proteins used in the split-luciferase assay. Numbers correspond to amino acid positions. (A) Ankyrin (ANK1). ZU5 corresponds to the zona occludens 1 (ZO-1) protein/unc5-like netrin receptor domain; DD, the death domain. The shaded region indicates hinge region following ankyrin repeats. (B) Band 4.1 (4.1R). FERM, Four-point-one, Ezrin, Radixin, Moesin domain (this region is also referred to as the 4.1R 30 kDa domain); SABD, the spectrin-actin-binding domain; and CTD, the C-terminal domain. (C) Band 4.2 (4.2R. Transglut_N, transglutaminase family; TGc, Transglutaminase/protease-like homologues; Transglut_C, Transglutaminase family, C-terminal immunoglobulin like

domain. (D) β-actin (ACTB). (E) Tropomyosin. (F) Glyceraldehyde 3-phosphate dehydrogenase (GAPDH) (G) β-spectrin (SPTB). (H) β-adducin (ADD2).

Supplementary Fig. S4. Domain organization of *P. falciparum* exported proteins that bound to erythrocyte cytoskeletal proteins. *P. falciparum* proteins are represented as rectangular bars whose length is proportional to the size in amino acids, as indicated by the scale bar. Black lines represent protein fragments used in the IOV binding and split-luciferase assays. Numbers flanking the black lines correspond to amino acid positions of the fragment. The PEXEL motif is immediately upstream of the fragment start site.

Shakya et al Supplementary Fig. S1

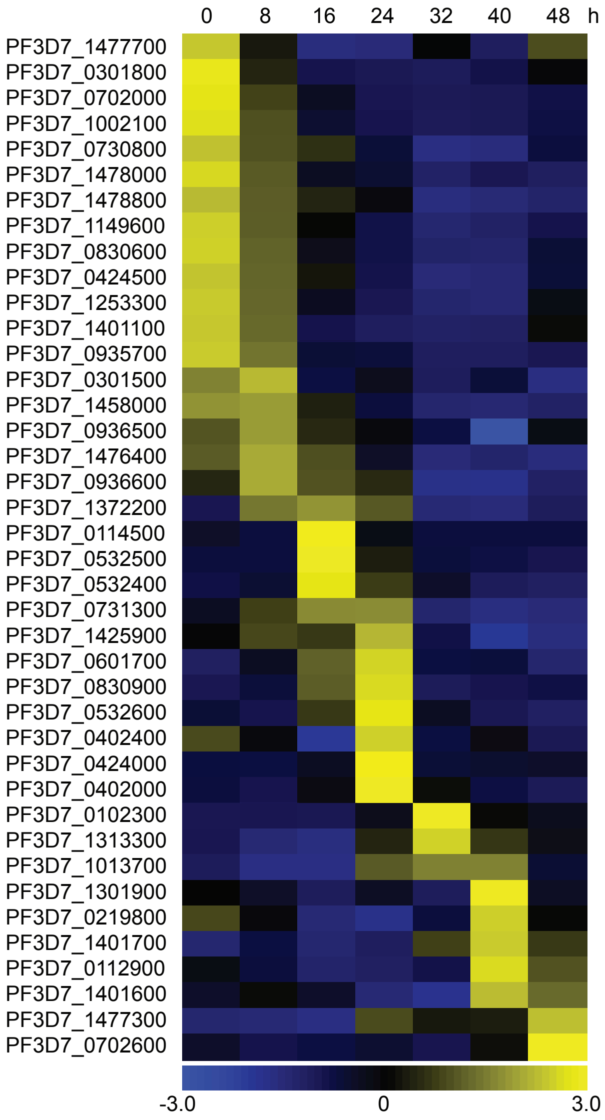

Shakya et al.
Supplementary Fig. S2

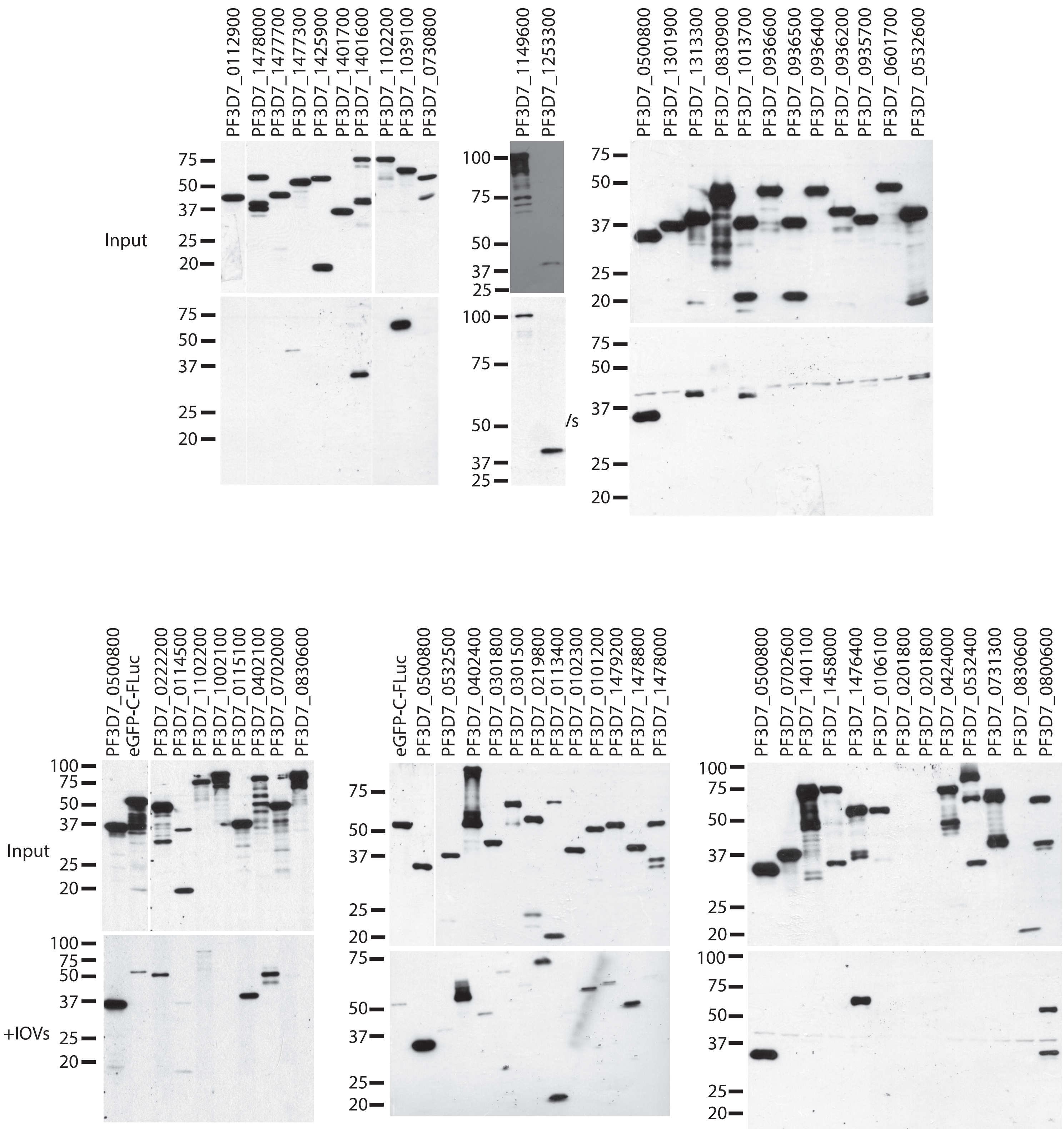

Shakya et al.
Supplementary
Fig. S3

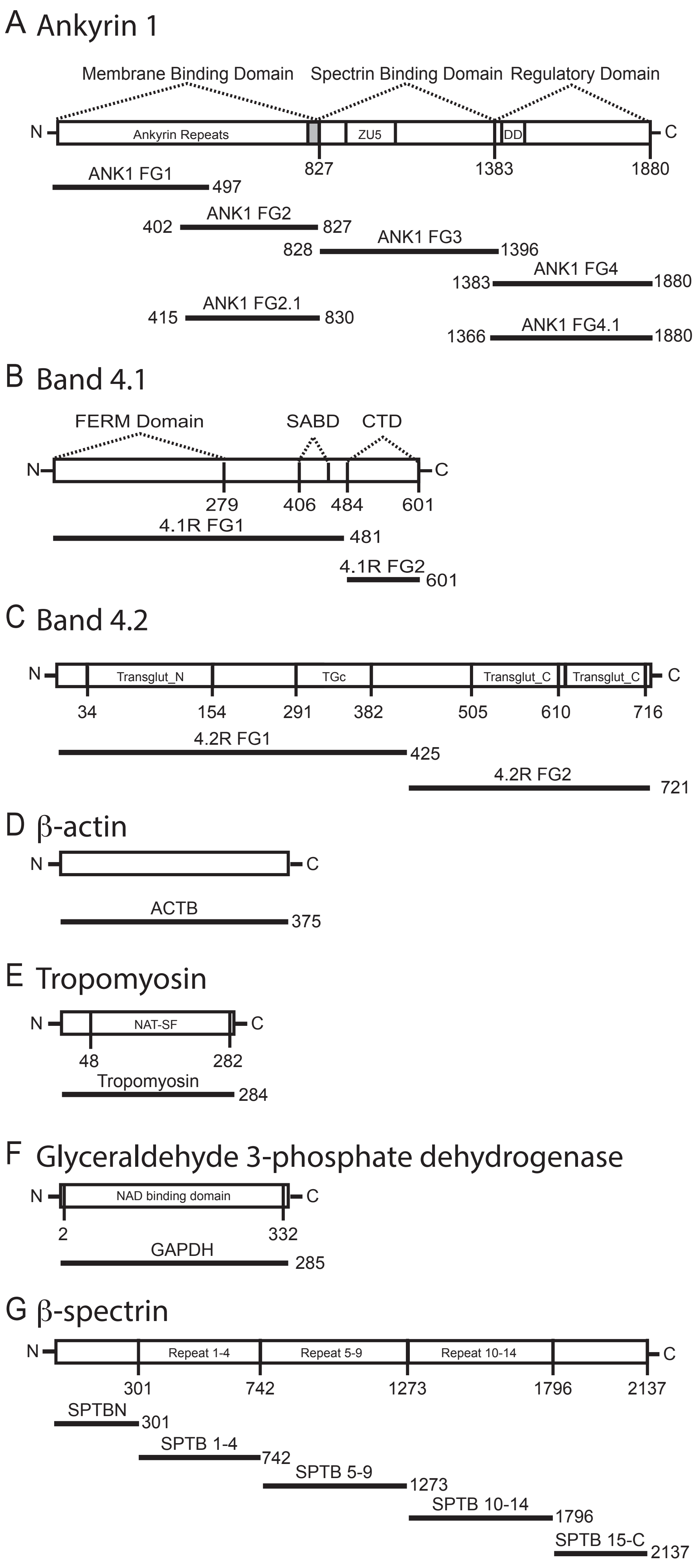

Shakya et al.
Supplementary Fig. S4

Domain organization of *P. falciparum* proteins shown in the protein-protein interaction map generated from the split-luciferase assay

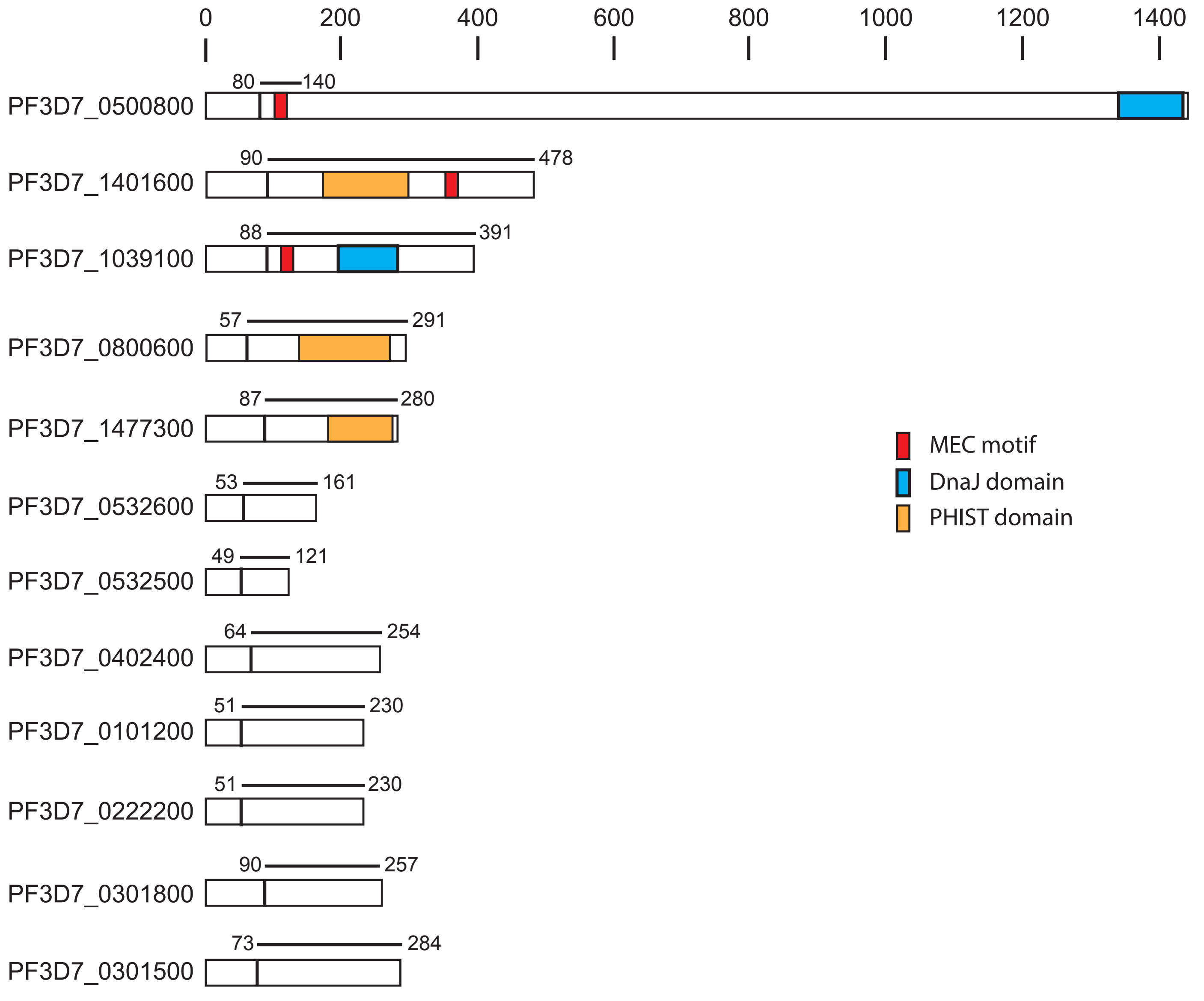

Supplement: Supplementary file 1 [file microorganisms-10-01438-s001.zip › microorganisms-1780029-supplementary/microorganisms-1780029-supplementary/Shakya Supplementary Figures.pdf]
